# The diagnostic value of blood metagenomic next-generation sequencing in patients with acute hematogenous osteomyelitis

**DOI:** 10.3389/fcimb.2023.1106097

**Published:** 2023-01-27

**Authors:** Bingshi Zhang, Xiao Chen, Xiaowei Yao, Mengnan Li, Zhijie Li, Bo Liu, Sikai Liu, Zeming Liu, Jia Huo, Yongtai Han

**Affiliations:** ^1^ Department of Osteonecrosis and Hip Surgery, The Third Hospital of Hebei Medical, Shijiazhuang, Hebei, China; ^2^ Department of Orthopedics, The Chest Hospital of Hebei Province, Shijizhuang, Hebei, China; ^3^ Orthopedics Department, Affiliated Hospital of Hebei Engineering University, Handan, Hebei, China

**Keywords:** diagnostic test, metagenomic next-generation sequencing (mNGS), infection, osteomyelitis (OM), blood

## Abstract

**Aims:**

This study aims to evaluate the diagnostic value of blood metagenomic next-generation sequencing (mNGS) in detecting pathogens from patients clinically diagnosed as acute hematogenous osteomyelitis (AHO).

**Methods:**

This retrospective study enrolled 66 patients with AHO. The test results of mNGS and bacterial culture on different samples, including blood and puncture fluid samples, from patients with AHO were compared to explore the diagnostic value of blood mNGS. Besides, this study also explored the efficacy of blood mNGS in decision making for antibiotic administration and analyzed the factors associated with the positive result of blood mNGS.

**Results:**

The most common causative pathogens were *Staphylococcus* and *Streptococcus*. The sensitivity of blood mNGS (77.3%) was higher than that of blood culture (42.4%) (*P*<0.001), while the turnaround time of blood mNGS (2.1 ± 0.4 d) is much less than that of blood culture (6.0 ± 2.1 d) (*P*<0.001). Besides, the sensitivity of blood mNGS tests (77.3%) was slightly lower than that of puncture fluid mNGS (89.4%). Furthermore, detection comparison at pathogen level unravels that blood mNGS might be suitable for diagnosing AHO caused by common pathogens, while puncture fluid mNGS could be considered as preferred examination in diagnosing AHO caused by uncommon pathogens. Finally, three independent factors associated with the true positive result of blood mNGS in patients with AHO were identified, including Gram-positive pathogens (OR=24.4, 95% CI = 1.4-421.0 for *Staphylococcus*; OR=14.9, 95%CI= 1.6-136.1 for other Gram-positive bacteria), body temperature at sampling time (OR=8.2, 95% CI = 0.6-107.3 for body temperature of >38.5°C; OR=17.2, 95% CI = 2.0-149.1 for patients who were chilling), and no use of antibiotics before sampling (OR=8.9, 95% CI =1.4-59.0).

**Conclusion:**

This is the first report on evaluating and emphasizing the importance of blood mNGS in diagnosing AHO. Blood sample might be an alternative sample for puncture fluid for mNGS, and its extensive application in diagnosing AHO could be expected.

## Introduction

Generally, based on the typical clinical manifestation, such as high fever, local tenderness, and elevated erythrocyte sedimentation rate (ESR) and C-reactive protein (CRP) level, the diagnosis of acute hematogenous osteomyelitis (AHO) is easy to be established (Funk and Copley, 2017). Without rapid etiological diagnosis and proper therapy, AHO may progress to chronic osteomyelitis (Alvares and Mimica, 2020; Wang and Zhang, 2022; Donders et al., 2022) which is difficult to be cured, or may even result in amputation. Therefore, the early diagnosis and treatment of AHO is crucially important for improving the prognoses of patients. However, how to rapidly and accurately identify the infectious pathogens is still a challenging problem that orthopaedic surgeons have to face seriously, since a significant part of AHO are not caused by *Staphylococcus aureus (* Castellazzi et al., 2016).

Bacterial culture is a commonly used method for pathogen detection. The samples used for culture could be blood, puncture fluid, or debridement fluid samples. However, low sensitivity limits its extensive application in accurate diagnosis and treatment. Alex et al. reported that the sensitivity of blood culture in patients with AHO was only 10-50% (Gornitzky et al., 2020). Although the sensitivities of puncture fluid culture or debridement fluid culture were relatively higher, the sampling processes are invasive, and the turnaround time is relatively long. In addition, during this period, the empirical antibiotic treatment might be ineffective, significantly affecting the prognoses of patients.

Therefore, how to rapidly and accurately determine the infectious pathogens is still demanded. Metagenomic next-generation sequencing (mNGS), allowing for high throughput DNA sequencing of pathogen nucleic acid, has been widely used for the detection of microbes and found to be a highly efficient tool for the identification of pathogens. mNGS showed more obvious advantages than culture in detecting pathogens, especially when bacterial load is low, significantly empowering the accurate diagnosis of related diseases (Fang et al., 2022). Some studies had demonstrated that the mNGS of pathogen-rich puncture fluid samples could help in detecting pathogens from patients with AHO (Ramchandar et al., 2021). For example, Zhang et al. found that cryptococcal osteomyelitis could be diagnosed by mNGS when the culture was negative (Zhang et al., 2019). Besides, mNGS showed slightly greater advantage in detecting pathogens from osteoarticular infection samples than conventional culture (88.5% vs 69.2%, *p* < 0.01), especially for the patients having previously received antibiotic treatment (Huang et al., 2020). Given blood sampling is noninvasive and pathogens of AHO are derived from blood, it raises a question that whether blood mNGS can accurately diagnose AHO. However, there are few researches on investigating the performance of blood mNGS in detecting pathogens from patients with AHO.

For infectious diseases in other systems, the advantages of blood mNGS in pathogen detection have already been demonstrated. For instance, Chen et al. reported that bloodstream infection in patients with severe pneumonia could be rapidly diagnosed by blood mNGS (Chen et al., 2021). Besides, Nan et al. also found that blood mNGS was an effective method to identify microorganisms from immunocompromised children with bloodstream infections (BSI) (Nan et al., 2022). Hence, we hypothesized that AHO could be diagnosed by blood mNGS.

To verify our hypothesis, this retrospective study was conducted to evaluate the performance of blood mNGS in detecting pathogens from patients clinically diagnosed as AHO against culture and puncture fluid mNGS. The main research questions are: (1) to obtain the sensitivity of blood mNGS in patients with AHO; (2) to determine whether blood mNGS could correctly identify the infectious pathogens; (3) to dig out which factors may influence the positive rate of blood mNGS.

## Material and methods

### Patient recruitment

The patients admitted to the Third Hospital of Hebei Medical University from January 1, 2021, to May 31, 2022 and diagnosed as AHO were included in the study. The clinical diagnosis of AHO was based on the clinical practice guideline of the Pediatric Infectious Diseases Society and the Infectious Diseases Society of America (IDSA). Medical history, clinical manifestation, laboratory examination, and imaging diagnosis were summarized. Inclusion criteria of patients were as follows: (1) acute osteomyelitis, with infection duration prior to treatment less than two weeks; (2) patient who undergone blood culture and mNGS; and (3) patients with definite bacteriological diagnosis, which was established *via* both blood sampling, and local puncture sampling or debridement surgery. Exclusion criteria of the patients were as follows: (1) patients with chronic recurrent multifocal osteomyelitis [CRMO] or Bacillus Calmette Guerin osteomyelitis; (2) patients with long term antibiotic medication therapy before osteomyelitis onset; and (3) lack of definite clinical or bacteriological diagnosis, namely, suspected osteomyelitis or undetermined bacteriological diagnosis.

### Sample size and study design

The sample size was estimated prior to the inclusion of these patients. The StatBox (https://www.biostats.cn/statbox/), an online statistical computing system, was used for estimation of sample size. It was assumed that the sensitivities of blood culture and blood mNGS were 0.4 and 0.75, respectively. When the significance level and power of the test were set to 0.05 and 0.8, respectively, the number of needed patients was 31 using Pearson chi-square estimation. To ensure the accuracy of data produced, we finally included 66 patients in our study.

According to the aims of this study, we summarized the performance of culture and mNGS tests using blood and puncture fluid samples. Besides, the study also explored the efficacy of blood mNGS in making decision for antibiotic administration and the factors associated with the positive rate of blood mNGS.

### Ethics statement

This study conducted in accordance with the Declaration of Helsinki was approved by the Institutional Review Board of the Third Hospital of Hebei Medical University. Because this was a retrospective study and the information of all patients was deidentified before analysis, this exemption from requiring informed consent was granted by the Ethics Committee of the Third Hospital of Hebei Medical University.

### Sample collection

Blood and puncture fluid samples of each patient were collected and further used for pathogen detection by both mNGS and bacterial culture. The acquisition of samples was conducted following a standard operating procedure (SOP). It was briefly described here: The sampling was performed in a treatment room, which was disinfected using ultraviolet light for at least 1 hour before sampling. Median cubital vein was selected as blood sampling sites, which were sterilized at least three times (15 cm around the puncture point) with cotton balls dipped with 0.2% iodine and 75% ethanol. Tourniquet was fastened before the skin puncture and released after the success of puncture. And then the blood sample was saved in a sterilized vacuum blood collection tube without anti-coagulation.

After the suspected diagnosis establishment, the first blood sampling was immediately performed and then at two-hour intervals thereafter. This blood sample acquisition was conducted three times on each patient. The collected samples were divided into two parts. One was used for mNGS detection and the other was used for bacterial culture and antibiotic susceptibility test. Note that, the first blood sampling was conducted before antibiotic treatment in our institute, while the antibiotic treatment was immediately performed after the first sampling.

Puncture fluid samples were collected at the site with the most obvious tenderness by layered sampling method within 72 hours of patients’ admission. This was also performed in the same treatment room. The local skin was sterilized at least three times (15 cm around the puncture point) with cotton balls dipped with 0.2% iodine and 75% ethanol, and 1% lidocaine was used for local anaesthesia. Skin was punctured using a pin with a diameter of 2 mm and the fluid was drawn from the soft-tissue and cancellous bone. The samples were also divided into two parts, for mNGS detection and bacterial culture.

### Clinical data collection

Clinical data was extracted from medical records, including demographic characteristic (age and sex), clinical information, laboratory test results (blood cell count, CRP, ESR, procalcitonin (PCT)), and detection of infectious pathogens. Body temperature recorded at the first blood sampling was collected for further analysis. Fasting vein blood samples were routinely collected on the first morning of hospitalization for laboratory test, the results of which were recorded in the study. In addition, the administration of antibiotics before admission was summarized for each patient.

### mNGS

The process of mNGS was described as follows: (1) DNA extraction: DNA from blood and puncture fluid samples was extracted using the Tianamp micro DNA Kit (DP316, Tiangen Biotech, Beijing, China) according to the manufacturer’s instructions. (2) Construction of DNA libraries and sequencing: DNA library construction included fragmenting DNA, repairing end-performing phosphorylation, ligating adaptor, and amplifying polymerase chain reaction (PCR) amplification. The quality of DNA library was evaluated using Qubit (Thermo Fisher Scientific, MA, USA) and Agilent 2100 Bioanalyzer (Agilent Technologies, Santa Clara, CA). All qualified libraries were sequenced on Nextseq 550 platform (Illumina, San Diego, USA). (3) Bioinformatics analysis: The high-quality sequencing data was obtained after removing low-quality and short (length < 35 bp) reads and following eliminating human host sequences. Such high-quality data was finally blasted against microbial databases, including bacterial, viral, fungal, and parasitic databases.

### Diagnostic value of mNGS

In this study, the clinical bacteriological diagnosis determined by clinical surgeons according to the clinical manifestation, laboratory examination, imaging diagnosis, culture and mNGS results of different samples, the treatment effect of antibiotics, and clinical experiences, was set as “gold standard” to evaluate the accuracy of mNGS. The primary index of the study was the sensitivity of blood mNGS. The secondary indexes included misdiagnosis rate and diagnosis time.

As mentioned above, blood sample acquisition was conducted three times on each patient and the three blood samples were all used for culture, while only the first blood sample collected was used for mNGS test. Both puncture fluid mNGS and culture were performed once. Positive results of mNGS or culture were defined as that the infectious pathogen was correctly determined by mNGS or culture, while misdiagnosis results were defined as that the results of mNGS or culture were inconsistent with clinical bacteriological diagnosis (‘gold standard’ above). For blood mNGS, puncture fluid mNGS, and puncture fluid culture, sensitivity and misdiagnosis rate are the numbers of positive results and misdiagnosis results divided by the number of enrolled patients, respectively. For blood culture, the numbers of positive results and misdiagnosis results divided by the total number of blood culture performed on the enrolled patients are sensitivity and misdiagnosis rate, respectively. Diagnosis time was identified as the time period from sampling to result report. Besides, the factors which may influence the positive result of blood mNGS were analyzed.

### Statistical analysis

Continuous variables were expressed as the mean ± standard deviation [SD], while the categorical variables were presented as count (proportion) when appropriate. Sensitivity, total sensitivity, misdiagnosis rate, and diagnosis time were compared between mNGS and bacterial culture on different samples by applying Pearson’s chi-squared test, McNemar test and paired *t*-test as appropriate. Logistic regression was used for analyzing the factors which may influence the positive result of blood mNGS. A *P* value less than 0.05 was considered to be significant. The above statistical analyses were conducted by using SPSS 19.0 statistical software for Windows (IBM, Armonk, NY) and Excel 2022 for Windows (Microsoft Corporation, Seattle, WA).

### Data availability

Sequencing data were deposited to the National Genomics Data Center under accession number PRJCA013362. The authors declare that the main data supporting the findings are available within this article. The other data generated and analyzed for this study are available from the corresponding author upon reasonable request.

## Result

### Demographic and clinical features of patients

A total of 66 patients diagnosed with AHO were enrolled in the current study. The median age of the enrolled patients was 10 years old, and 37 (56.1%) patients were male (**Table 1**). The most commonly infected bone was distal femur (*n* =29, 43.9%), followed by proximal tibia (*n* =26, 39.4%). The mean follow-up period was 18.7 ± 3.8 months. The infection parameters of patients with AHO were assessed (**Table 2**). In addition to fever, some AHO patients had other symptoms, including chills, soft tissue erythema, swollen and draining sinus tracts, *etc.* The blood testing of patients with AHO showed the classic manifestation of bacterial infection. Blood cell counts such as leukocytes ((10.8 ± 1.9) ×10^9^/L) and neutrophils ((8.2 ± 2.0) ×10^9^/L, 75.0 ± 6.5%) were at relatively high level. Moreover, the CRP was at a high level (61.0 ± 35.3 mg/L), as was the ESR (63.0 ± 23.2 mm/h). The mean time period from the onset of symptom to the first blood sampling was 77.6 ± 31.0 hours. More than half of the patients (*n* = 37) developed fever or chilling. The temperatures of 17 (25.8%) and 20 (30.3%) patients at the time of sampling were < 38.5°C and ≥38.5°C, respectively, while 29 patients were chilling (43.9%). In addition, 13 patients (19.7%) received antibiotic treatment before sampling (**Table 2**).

**Table 1:** Demographic characteristics and clinical information of the patients with AHO.

Characteristics	Number of cases
Age (years)	
1-3 years	7
4-6 years	12
7-12 years	28
13-16 years	13
>16 years	6
Sex	
Male	37
Female	29
**Weight (kg)**	37.6 ± 17.3
**Body mass index**	19.6 ± 8.8
Affected bone	
Distal femur	29
Proximal tibia	26
Other metaphysis of long tube bone	5
Shaft of long tube bone	4
Other	2
**Follow-up time (months)**	18.7 ± 3.8

**Table 2:** Infectious parameters in patients with AHO.

Parameters	
**Time period from the onset of symptom to first blood sampling (hours)**	77.6 ± 31.0
Body temperature at sampling	
<38.5°C	17
≥38.5°C	20
Chilling	29
Antibiotics administration before admission (*n*)	
No	53
Yes	13
**Average weighted temperature (degree of centigrade)**	38.5
Blood cell count	
Leukocytes	10.8 ± 1.9
Hemoglobin (g/L)	136.9 ± 12.2
Neutrophils	8.2 ± 2.0
Neutrophils, %	75.0 ± 6.5
Lymphocytes	1.9 ± 0.4
Monocytes	0.4 ± 0.2
Platelets	298.8 ± 29.0
**C-reactive protein (mg/L)**	61.0 ± 35.3
**Erythrocyte sedimentation rate (mm/h)**	63.0 ± 23.2
**Procalcitonin (ng/mL)**	0.2 ± 0.1
Infectious bacteria (*n*)	
*Acinetobacter*	3
*Brucella*	1
*Mycobacterium tuberculosis*	1
*Enterococcus*	6
*Enterobacteriaceae*	2
*Haemophilus influenzae*	3
*Klebsiella*	7
*Pseudomonas aeruginosa*	7
*Staphylococcus*	19
*Streptococcus*	17

### Pathogen profile

Combined with the clinical manifestation, laboratory examination, imaging diagnosis, culture, mNGS results, and clinical experience, we defined causative pathogens of AHO (**Table 2**). It was found that all of the causative pathogens were bacteria, of which Gram-positive bacteria accounted for 63.6%. The common causative pathogens were *Staphylococcus* (*n* = 19) and *Streptococcus* (*n* = 17), followed by *Klebsiella* (*n* = 7), *Pseudomonas aeruginos*a (*n* = 7), *Enterococcus* (*n* = 6), *Acinetobacter* (*n* = 3), *Haemophilus influenzae* (*n* = 3), and *Escherichia* (*n* = 2). Besides, *Brucella* (*n* = 1) and *Mycobacterium tuberculosis* (*n* = 1) were also proven to cause AHO.

### Comparison between blood mNGS and culture

Compared with conventional blood culture, blood mNGS can accurately detect more pathogens (**Table 3**, **Figure S1**). The sensitivity of blood mNGS was 77.3%, which was higher than that of blood culture (42.4%) (*P <*0.001). There was no significant difference in the misdiagnosis rate between blood mNGS and blood culture (6.1% *vs* 3.5%, *P*=0.474). However, the turnaround time of mNGS is much less than that of blood culture (2.1 ± 0.4 *vs* 6.0 ± 2.1 days, *P*<0.001).

**Table 3:** Comparison of mNGS diagnostic value between blood and puncture samples.

Diagnostic parameters	Blood sample				Puncture fluid sample			
	mNGS	Bacterial culture	Test statistics	*P*	mNGS	Bacterial culture	Test statistics	*P*
Sensitivity (First sampling)	77.3%	42.4%	16.7	<0.001	89.4%	87.9%	0.08	0.784
Total Sensitivity (First three samplings)	–	63.6%	–	–	–	–	–	–
Misdiagnosis (%)	6.1%	3.5%	–	0.474	4.7%	1.5%	–	0.619
Diagnosis time (days)	2.1 ± 0.4	6.0 ± 2.1	-10.8	<0.001				

### Advantages of blood mNGS and puncture fluid mNGS

To evaluate the performance of blood mNGS in diagnosing AHO, puncture fluid sample was simultaneously collected for mNGS test (**Figure S2**). It was found that the sensitivity of blood mNGS (77.3%) was slightly lower than that of puncture fluid mNGS (89.4%) and puncture fluid culture (87.9%) (**Table 3**). Besides, as focal sample, misdiagnosis rates of puncture fluid mNGS (4.65%) and puncture fluid culture (1.5%) were slightly lower than that of blood mNGS (6.1%). High performance and low misdiagnosis rate indicate that blood sample might be an alternative sample for puncture fluid for mNGS in diagnosing AHO.

### Pathogen detection by different methods

**Figure 1:**
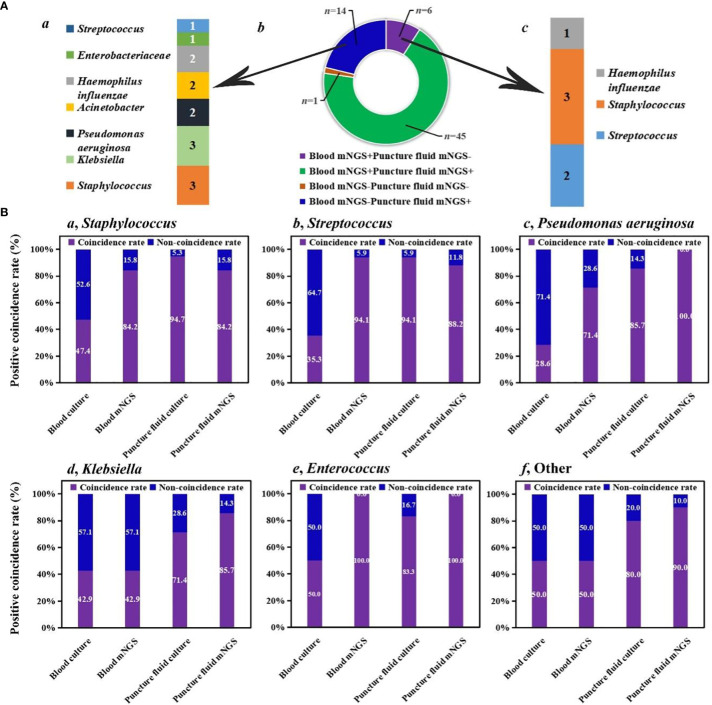
Comparison among different methods. **(A)** Detection performance of blood and puncture fluid mNGS. **(B)** Coincidence rates of different methods against final clinical diagnoses.

We investigated the detection performance of pathogens by blood mNGS, blood culture, puncture fluid mNGS, and puncture fluid culture (**Figure 1**). Blood mNGS and puncture fluid mNGS can simultaneously detect pathogens from most of the enrolled patients (*n* =45), while there was only 1 patient with both blood and puncture fluid mNGS negative. For the patients (*n* =14) with blood mNGS negative (-) and puncture fluid mNGS positive (+), *Staphylococcus* (*n* =3) and *Klebsiella* (*n* =3), *Pseudomonas aeruginosa* (*n* =2), *Acinetobacter* (*n* =2), *Haemophilus influenzae* (*n* =2) were detected. While for the patients with blood mNGS positive (+) and puncture fluid mNGS negative (-), *Streptococcus* (*n* =2) and *Staphylococcus* (*n* =3) were identified. The above results indicate that blood mNGS might be much more suitable for detecting common pathogens of AHO, rather than uncommon pathogens.

We further summarized positive coincidence rates of different methods at the pathogen level. For *Staphylococcus*, while the positive coincidence rate of blood culture (47.4%) was less than 50%, 84.2% of results revealed by blood mNGS were consistent with final clinical diagnoses. Besides, most of *Staphylococcus* can be detected by puncture fluid culture (94.7%), and the positive coincidence rate of puncture fluid mNGS was the same as that of blood mNGS. A same trend was found in the detection of *Streptococcus*. Interestingly, positive coincidence rate of blood mNGS (94.1%) for *Streptococcus* was higher than that of puncture fluid mNGS (88.2%).

For *Pseudomonas aeruginosa*, positive coincidence rate of puncture fluid mNGS can reach up to 100%, higher than that of blood mNGS (71.4%). Puncture fluid culture can successfully detect *Pseudomonas aeruginosa* from 85.7% of patients, while only 28.6% of patients can be accurately diagnosed by blood culture. Same trends were also found in the detection of *Klebsiella*, *Enterococcus*, and other uncommon pathogens of AHO. The above results provide explanation for our finding that blood mNGS was suitable for detecting common pathogens of AHO, and further unravel that blood and puncture fluid sample might be considered as preferred sample for mNGS in detecting AHO caused by common and uncommon pathogens, respectively.

### Factors associated with mNGS sensitivity

Three independent factors identified were associated with the true positive result of blood mNGS in patients with AHO, including detected pathogens, body temperature at sampling time, and antibiotic administration before sampling (**Table 4**). Compared with Gram-negative bacteria, the estimated odds ratio for *Staphylococcus* was 24.4 (95% CI = 1.4-421.0, *P*=0.028) in this cohort of patients with AHO, and it was 14.9 (95% CI = 1.6-136.1, *P*=0.017) for other gram-positive bacteria. Another independent factor was body temperature at sampling. Compared with body temperature of <38.5°C, the estimated odds ratio for body temperature of ≥38.5°C and chilling were 8.2 (95% CI = 0.6-107.3, *P*=0.111) and 17.2 (95% CI = 2.0-149.1, *P*=0.010), respectively, indicating that higher body temperature or chilling may cause a rise in mNGS sensitivity. Besides, compared with antibiotic administration before sampling, the estimated odds ratio for the patients without antibiotic treatment was 8.9 (95% CI = 1.4-59.0, *P*=0.023), suggesting that antibiotic treatment before sampling may cause a decrease in the positive rate of mNGS.

**Table 4:** Factors associated with the positive results of mNGS in patients with AHO.

Factors	Odds ratio	95% confidence interval for odds ratio	*P*
Infected bacteria			
*Staphylococcus*	24.4	1.4-421.0	0.028
Other Gram-positive bacteria	14.9	1.6-136.1	0.017
Other Gram-negative bacteria	Ref.		
Body temperature at sampling			
Chilling	17.2	2.0-149.1	0.010
≥38.5°C	8.2	0.6-107.3	0.111
<38.5°C	Ref.		
Antibiotics administration before sampling			
Yes	Ref.		
No	8.9	1.4-59.0	0.023

## Discussion

To the best of our knowledge, this is the first report on evaluating the performance of blood mNGS in patients with AHO. The common causative pathogens of AHO were *Staphylococcus* and *Streptococcus.* Taking final clinical diagnoses as gold standard, we found that the sensitivity of blood mNGS (77.3%) was slightly lower than that of puncture fluid mNGS (89.4%). Furthermore, detection comparison at pathogen level unravels that blood mNGS might be suitable for diagnosing AHO caused by common pathogens, while puncture fluid mNGS could be considered as preferred examination in diagnosing AHO caused by uncommon pathogens.

We proved that blood mNGS could be used for diagnosing AHO, challenging the long-held opinion that puncture fluid sample is essential for mNGS test in detecting pathogens of AHO. For conventional methods, the most suitable sample for infection diagnosis is focal sample, while low or zero pathogen loads in blood limit its extensively application in accurate infection diagnosis. Haessler et al. found that *Streptococcus pneumoniae* (33%) and *S. aureus* (22%) causing severe pulmonary infection can be successfully isolated from blood samples, indicating that pathogens in focal site spread to bloodstream and blood sample might be considered as an alternative sample for diagnosing severe pulmonary infection (Haessler et al., 2020). While for hematogenous infection, the clinical problem is whether the microbes detected from blood samples are causative pathogens, rather than whether the microbes could be detected. Rakow et al. found that more than 61% of pathogen (43/70) causing hematogenous periprosthetic joint infection can be successfully detected from blood samples (Renz et al., 2022). For AHO, we found that the coincidence rate (sensitivity) of blood mNGS tests (77.3%) against final clinical diagnosis was slightly lower than that of puncture fluid mNGS (89.4%). Interestingly, positive coincidence rate of blood mNGS (94.1%) for *Streptococcus* was higher than that of puncture fluid mNGS (88.2%), which might be because 1) the *Streptococcus* was not collected from the focal site during the puncture fluid sampling or 2) *Streptococcus* load in some puncture fluid sample was too low to be detected by mNGS. According to our results, we propose that blood sample might be an alternative sample of puncture fluid for mNGS in diagnosing AHO.

In addition, the diagnosis time of blood mNGS (2.1 ± 0.4 d) was significantly shorter than that of blood culture (6.0 ± 2.1 d) in this study. Grumaz et al. reported that mNGS detection only took 30 h (turnaround time) to accomplish the whole process from blood sample preparation to pathogens identification report (Grumaz et al., 2019). For culture, although the growth time of Gram-negative bacteria and Gram-positive bacteria is 11 h (10-13 h) and 12 h (12-18 h), respectively, the turnaround time of bacterial culture is more than 7 d, which is much longer than growth time (Mendoza et al., 2015). Therefore, mNGS could be considered to be a promising tool for guiding the timely and accurate use of antibiotics.

Finally, this study explored the influence factors associated with positive result of mNGS, which may be crucial for reasonable interpretation of results and clinical application. An early study suggested that a blood culture was more likely to show positive results if it was taken prior to a temperature spike, which was further confirmed by the study of Kee et al. that bacteremia preceded a fever (Kee et al., 2016). We also found that higher body temperature or chilling may cause a rise in mNGS sensitivity. Moreover, previous study reported that the mNGS performance is less likely to be affected by prior use of antibiotics, which was contrary to the results of our study (Miao et al., 2018). In the treatment of osteomyelitis, β-lactams were listed as the first choice of antibiotic usage (Nade, 1977; Autore et al., 2020). It was found that β-lactams can kill pathogen *via* destroying their cell walls, decreasing the abundance of pathogen DNA in bloodstream and resulting in negative result of subsequent mNGS detection. Understanding factors associated with the positive result of blood NGS could potentially promote the clinical diagnostic value of blood mNGS.

The limitations of this study are as follows. (1) The current sample size is relatively small and further studies should enroll more patients to generate more accurate findings. (2) The study was conducted by retrospective analysis, which may limit the comprehensive analysis or result in information bias. (3) The analysis of mNGS cost was not performed, which may limit its clinical application.

## Conclusions

The study demonstrated that blood mNGS did exhibit good performance in identifying the pathogens from patients with AHO. Taking final clinical diagnoses as gold standard, the sensitivity of blood mNGS (77.3%) was slightly lower than that of puncture fluid mNGS (89.4%). Furthermore, blood mNGS and puncture mNGS might be suitable for diagnosing AHO caused by common pathogens and uncommon pathogens, respectively. Besides, antibiotic treatment before sampling may cause a decrease in the positive rate of mNGS.

## Data availability statement

The datasets presented in this study can be found in online repositories. The names of the repository/repositories and accession number(s) can be found in the article/**Supplementary Material**.

## Ethics statement

Because this was a retrospective study and the information of all patients was identified before analysis, this exemption from requiring informed consent was granted by the Ethics Committee of the Third Hospital of Hebei Medical University. Written informed consent to participate in this study was provided by the participants’ legal guardian/next of kin.

## Author contributions

YH designed the paper. BZ and XC drafted the manuscript. XY, ML, and ZhL carried out the clinical care and management of the patients. BL and BZ performed the mNGS tests and analyzed the data. ZeL and JH revised the manuscript. All authors contributed to the article and approved the submitted version.
