# Supplementary material for: The diagnostic value of blood metagenomic next-generation sequencing in patients with acute hematogenous osteomyelitis
Source: Front Cell Infect Microbiol. 2023 Jan 27;13:1106097. doi: 10.3389/fcimb.2023.1106097 (PMC9911542; doi:10.3389/fcimb.2023.1106097)
Supplement: Supplementary file 1 [file DataSheet_1.docx]

**The diagnostic value of blood metagenomic next-generation sequencing in patients with acute hematogenous osteomyelitis**

Bingshi Zhang^1,a^, Xiao Chen^1,a^, Xiaowei Yao^2^, Mengnan Li^1^, Zhijie Li^3^, Bo Liu^1^, Sikai Liu^1^, Zeming Liu^1^, Jia Huo^1^, Yongtai Han^1,*^

^1^Department of Osteonecrosis and Hip Surgery, the Third Hospital of Hebei Medical, No.139 Ziqiang Road, Shijiazhuang, Hebei Province, P.R. China.

^2^Department of Orthopedics, the Chest Hospital of Hebei Province, 372 Shengli North Street, Shijizhuang, Hebei Province, P.R. China.

^3^Orthopedics Department, Affiliated Hospital of Hebei Engineering University, No. 81 Congtai Road, Congtai District, Handan, Hebei Province, P.R. China.

^a^These authors have contributed equally to this work and share first authorship.

*Correspondence: Yongtai Han, [hanyongtai@hebmu.edu.cn](mailto:hanyongtai@hebmu.edu.cn).

| 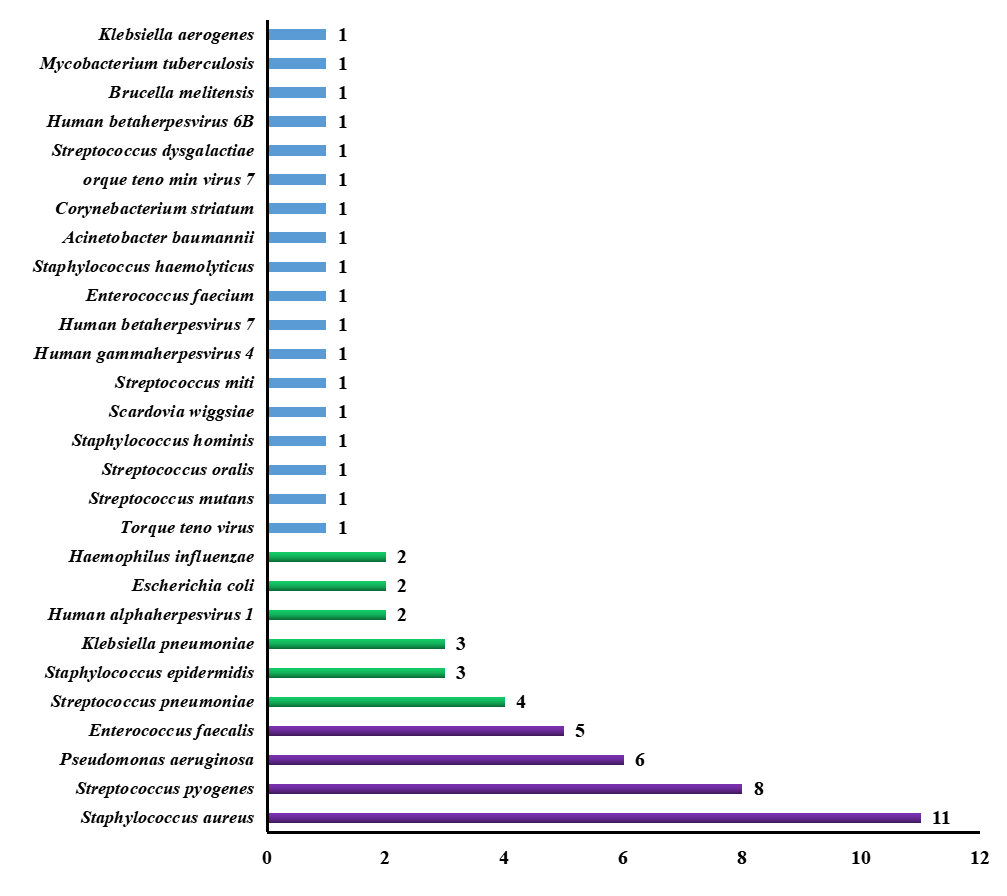 |
| --- |
| Figure S1 Microbes detected by blood mNGS |

| 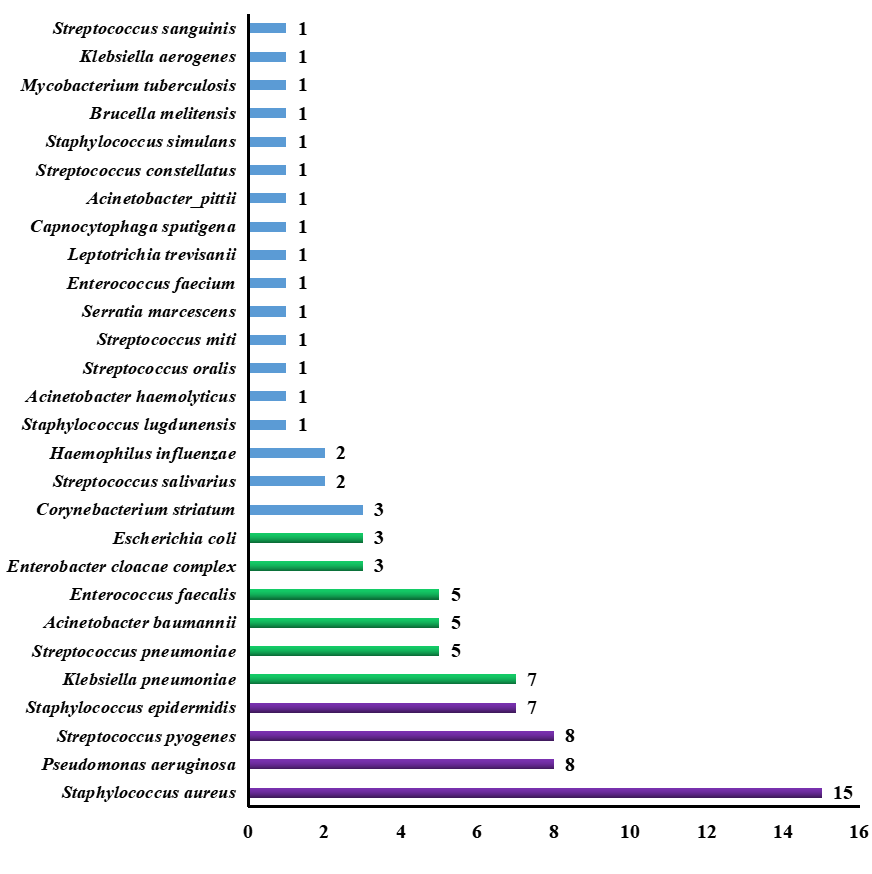 |
| --- |
| Figure S2 Microbes detected by puncture fluid mNGS |
